# Supplementary material for: The Synergistic Action of Metformin and Glycyrrhiza uralensis Fischer Extract Alleviates Metabolic Disorders in Mice with Diet-Induced Obesity
Source: Int J Mol Sci. 2023 Jan 4;24(2):936. doi: 10.3390/ijms24020936 (PMC9862386; doi:10.3390/ijms24020936)
Supplement: Supplementary file 1 [file ijms-24-00936-s001.zip › ijms-2097567-supplementary.pdf]

*Article*

# The synergistic action of metformin and *Glycyrrhiza uralensis Fischer* extract alleviates metabolic disorders in mice with diet-induced obesity

**Min-Kyeong Hong [1,2,3,#], Youngji Han [1,2,3,#], Hae-Jin Park [4,#], Mi-Rae Shin [5], Seong-Soo Roh [5,*] and Eun-Young Kwon [1,2,3,*]**

[1] Department of Food Science and Nutrition, Kyungpook National University, 1370 San-Kyuk Dong Puk-Ku, 41566 Daegu, Republic of Korea
[2] Center for Food and Nutritional Genomics Research, Kyungpook National University, 1370 San-Kyuk Dong Puk-Ku, 41566 Daegu, Republic of Korea
[3] Center for Beautiful Aging, Kyungpook National University, 1370 San-Kyuk Dong Puk-Ku, 41566 Daegu, Republic of Korea
[4] Bio Convergence Testing Center, Daegu Haany University, 1 Haanydaero, Gyeongsan-si, Gyeongsangbuk-Do, 38610 Daegu, Republic of Korea
[5] Department of Herbology, College of Korean Medicine, Daegu Haany University, 64 Gil, 25 Suseongro, Suseong-gu, 42158 Daegu, Republic of Korea
[#] These authors contributed equally to this work.
* Correspondence: Seong-Soo Roh, Ph.D. and Eun-Young Kwon, Ph.D.
Roh SS; Tel: +82-53-770-2258, Fax: +82-53-950-6229, E-mail: ddede@dhu.ac.kr
Kwon EY; Tel: +82-53-950-6231, Fax: +82-53-950-6229, E-mail: eykwon@knu.ac.kr

## Supplementary Material

**Table S1.** Diet composition for animal experiment I.

| Ingredient(g) | ND (AIN-93G) | HFD (60 kcal% fat) | LGU (0.015%) | HGU (0.03%) |
|---|---|---|---|---|
| **Casein** | 200 | 267 | 267 | 267 |
| **Corn starch** | 397.486 | 63.381 | 63.381 | 63.381 |
| **Sucrose** | 100 | 0 | 0 | 0 |
| **Dextrose** | 132 | 176 | 176 | 176 |
| **Cellulose** | 50 | 67 | 67 | 67 |
| **Soybean Oil** | 70 | 33 | 33 | 33 |
| **Lard** | 0 | 327 | 327 | 327 |
| **Mineral mixture** | 35 | 47 | 47 | 47 |
| **Vitamin mixture** | 10 | 13 | 13 | 13 |
| **TBHQ, antioxidant** | 0.014 | 0.019 | 0.019 | 0.019 |
| **L-cystine** | 3 | 4 | 4 | 4 |
| **Choline Bitartrate** | 2.5 | 3 | 3 | 3 |
| **GU** | - | - | 0.15 | 0.3 |
| **Total (g)** | **1000.00** | **1000.00** | **1000.15** | **1000.3** |
| **Calorie (kcal/g)** | **4000** | **5332.62** | **5332.62** | **5332.62** |

[1]AIN-93 mineral mixture (g/kg): calcium carbonate anhydrous, 357; potassium phosphate monobasic, 196; potassium citrate tripotassium monohydrate, 70.78; sodium chloride, 74; potassium sulfate, 46.60; magnesium oxide, 24; ferric citrate, 6.06; zinc carbonate, 1.65; sodium meta-silicate·9H2O, 1.45; manganous carbonate, 0.63; cupric carbonate, 0.30; chromium potassium sulfate·12H2O, 0.275; boric acid, 0.0815; sodium fluoride, 0.635; nickel carbonate, 0.0318, lithium chloride, 0.0174, sodium selenite anhydrous, 0.01025; potassium iodate, 0.010; ammonium paramolybdate·4H2O, 0.00795; ammonium vanadate 0.0066; powdered sucrose, 221.026 [2]AIN-93G- Vitamin Mixture (g/kg): nicotinic acid, 3; Ca

pantothenate, 1.6; pyridoxine-HCl, 0.7; thiamin-HCl, 0.6; riboflavin, 0.6; folic acid, 0.2; biotin, 0.02; vitamin B-12 (cyanocobalamin), 2.5; vitamin E (all-rac-$\alpha$-tocopheryl acetate), 15; vitamin A (all-trans-retinyl palmitate), 0.8; vitamin D-3 (cholecalciferol), 0.25; vitamin K-1 (phylloquinone), 0.075; powdered sucrose, 974.655. ND, Normal diet (AIN-93G 16 Kcal% fat); HFD, High fat diet (60 Kcal% fat); LGU, HFD + 0.015% *Glycyrrhiza uralensis Fischer* 0.015%; HGU, HFD + 0.003% *Glycyrrhiza uralensis Fischer* 0.015%;

**Table S2.** Diet composition for animal experiment Ⅱ.

| Ingredient(g) | ND | HFD | HFDM | GU | GUM |
|---|---|---|---|---|---|
| **Casein** | 200 | 267 | 267 | 267 | 267 |
| **Corn starch** | 397.486 | 63.381 | 63.381 | 63.381 | 63.381 |
| **Sucrose** | 100 | 0 | 0 | 0 | 0 |
| **Dextrose** | 132 | 176 | 176 | 176 | 176 |
| **Cellulose** | 50 | 67 | 67 | 67 | 67 |
| **Soybean Oil** | 70 | 33 | 33 | 33 | 33 |
| **Lard** | 0 | 327 | 327 | 327 | 327 |
| **Mineral mixture[1]** | 35 | 47 | 47 | 47 | 47 |
| **Vitamin mixture[2]** | 10 | 13 | 13 | 13 | 13 |
| **TBHQ, antioxidant** | 0.014 | 0.019 | 0.019 | 0.019 | 0.019 |
| **L-cystine** | 3 | 4 | 4 | 4 | 4 |
| **Choline Bitartrate** | 2.5 | 3 | 3 | 3 | 3 |
| **Metformin** | - | - | 0.5 | - | 0.5 |
| **GU** | - | - | - | 0.15 | 0.15 |
| **Total (g)** | 1000.00 | 1000.00 | 1000.50 | 1000.15 | 1000.65 |
| **Calorie (kcal/g)** | 4000 | 5332.62 | 5332.62 | 5332.62 | 5332.62 |

[1]AIN-93 mineral mixture (g/kg): calcium carbonate anhydrous, 357; potassium phosphate monobasic, 196; potassium citrate tripotassium monohydrate, 70.78; sodium chloride, 74; potassium sulfate, 46.60; magnesium oxide, 24; ferric citrate, 6.06; zinc carbonate, 1.65; sodium meta-silicate·9H2O, 1.45;

manganous carbonate, 0.63; cupric carbonate, 0.30; chromium potassium sulfate·12H2O, 0.275; boric acid, 0.0815; sodium fluoride, 0.635; nickel carbonate, 0.0318, lithium chloride, 0.0174, sodium selenite anhydrous, 0.01025; potassium iodate, 0.010; ammonium paramolybdate·4H2O, 0.00795; ammonium vanadate 0.0066; powdered sucrose, 221.026 [2]AIN-93G- Vitamin Mixture (g/kg): nicotinic acid, 3; Ca pantothenate, 1.6; pyridoxine-HCl, 0.7; thiamin-HCl, 0.6; riboflavin, 0.6; folic acid, 0.2; biotin, 0.02; vitamin B-12 (cyanocobalamin), 2.5; vitamin E (all-rac-$\alpha$-tocopheryl acetate), 15; vitamin A (all-trans-retinyl palmitate), 0.8; vitamin D-3 (cholecalciferol), 0.25; vitamin K-1 (phylloquinone), 0.075; powdered sucrose, 974.655. ND, Normal diet (AIN-93G 16 Kcal% fat); HFD, High fat diet (60 Kcal% fat); HFDM, HFD + 50mg/kg Metformin; GU, HFD + 0.015% Glycyrrhiza uralensis Fischer 0.015%; GUM, HFD + 0.015% Glycyrrhiza uralensis Fischer +50mg/kg Metformin.
